# An Integrated Screening Study of Polyphenol Recovery from Olive Mill Wastewater Using Cationic Starch-Assisted Clarification and Adsorption Resin Purification

**DOI:** 10.3390/antiox15080946

**Published:** 2026-07-29

**Authors:** Nina Knezovic, Ajka Pribisalic, Barbara Soldo, Ivica Ljubenkov, Roberta Frleta Matas, Sanja Luetic, Katarina Jurcic, Mladenka Sarolic, Davorka Sutlovic, Zlatka Knezovic

**Affiliations:** 1 Teaching Institute for Public Health, Split-Dalmatia County, 21000 Split, Croatia; nina.knezovic@nzjz-split.hr (N.K.); sanja.luetic@nzjz-split.hr (S.L.); katarina.jurcic@nzjz-split.hr (K.J.); zlatka.knezovic@nzjz-split.hr (Z.K.); 2 Faculty of Health Sciences, University of Split, 21000 Split, Croatia; dsutlovic@fzz.unist.hr; 3 Department of Chemistry, Faculty of Science, University of Split, Ruđera Boškovića 33, 21000 Split, Croatia; barbara@pmfst.hr (B.S.); ivica.ljubenkov4@gmail.com (I.L.); 4 Department of Food Technology and Biotechnology, Faculty of Chemistry and Technology, University of Split, R. Boškovića 35, 21000 Split, Croatia; rfmatas@unist.hr (R.F.M.); mladenka.sarolic@ktf-split.hr (M.S.); 5 Department of Applied Pharmacy, School of Medicine, University of Split, 21000 Split, Croatia

**Keywords:** olive mill wastewater, hydroxytyrosol, phenolic compounds, cationic starch, coagulation–flocculation, adsorption resin, antioxidant activity, circular bioeconomy

## Abstract

Olive mill wastewater (OMW) is an environmentally problematic effluent but also a rich source of antioxidant phenolic compounds. This study evaluated an integrated recovery approach combining cationic starch clarification and adsorption resin purification for obtaining phenolic-rich fractions from Dalmatian OMW. A pooled OMW sample was characterized for conventional wastewater parameters and total phenolic content (TPC), clarified with four commercial cationic starches, and subsequently processed using Seplite LXA84, Seplite LXA88, Seplite LXA816, and Amberlite XAD4 adsorption resins. PrimePHASE 2545 at 1000 mg active substance L^−1^ provided the most favorable balance between matrix removal and phenolic retention, reducing suspended solids and organic load while preserving approximately 92% of the initial TPC. In the preliminary resin screening, Seplite LXA84 produced the most favorable ethanol eluate, with the highest phenolic recovery and strong antioxidant activity, particularly in FRAP and ORAC assays. HPLC profiling confirmed hydroxytyrosol as the dominant recovered phenolic compound, accompanied by tyrosol and selected phenolic acids. These findings support a two-stage OMW valorization approach in which cationic starch pretreatment improves the suitability of OMW for column processing, while resin adsorption produces phenolic-rich fractions with antioxidant activity that may serve as candidates for further purification, characterization, and application-oriented research.

## 1. Introduction

Olive oil mill wastewater (OMW) is an acidic dark-brown effluent characterized by a high organic load and a complex mixture of bioactive compounds, particularly phenolic compounds. Because of its phytotoxic and antimicrobial effects, the direct discharge of untreated OMW can adversely affect soil microbiota, aquatic ecosystems, and surface water quality [1,2]. Toxic effects on aquatic organisms have been reported even at relatively low concentrations, as low as 40 mg L^−1^, while soil application of untreated OMW may impair plant germination and early growth [3]. Given that, according to the estimates from the International Olive Oil Council, approximately 30 million m^3^ of OMW are generated annually in the Mediterranean region, the treatment and valorization of this waste stream remain important environmental and technological challenges [4].

At the same time, the phenolic fraction of OMW represents a valuable resource. Olive polyphenols, especially hydroxytyrosol, have been associated with antioxidant, anti-inflammatory, cardioprotective, and neuroprotective effects [5,6]. The relevance of olive polyphenols has also been recognized by the European Food Safety Authority (EFSA), which states that olive oil polyphenols contribute to the protection of blood lipids from oxidative stress when the product contains at least 5 mg of hydroxytyrosol and its derivatives per 20 g of olive oil [7]. Hydroxytyrosol has also shown photoprotective and anti-aging potential in skin-related models, supporting its possible use in cosmeceutical applications [8]. Thus, OMW should not only be considered a pollutant, but also a renewable raw material for obtaining antioxidant-rich phenolic extracts.

Recovering phenolics from OMW is technically challenging because the matrix is a complex, polydisperse system containing dissolved organic matter, colloids, suspended solids, and dispersed oil droplets. Efficient recovery usually requires an integrated process in which clarification precedes concentration or purification. The first phase includes physical pretreatment, known as conventional clarification, combining coagulation, flocculation, and sedimentation. 

Coagulation destabilizes particles by neutralizing their surface charge, while flocculation promotes the formation of larger flocs that are subsequently removed by sedimentation. Sedimentation alone, without prior coagulation and flocculation, removes only relatively coarse suspended solids [9]. Inorganic aluminum and iron salts are the most commonly used coagulants, as they neutralize the surface charge of OMW particles, whereas flocculants promote particle aggregation and facilitate their removal by sedimentation [10].

The most common flocculants are synthetic polyacrylamide-based polymers (anionic, nonionic, and cationic) [11,12]. Although classic inorganic coagulants and synthetic flocculants are highly effective in wastewater treatment, their use may raise environmental and health-related concerns. These include the generation of sludge rich in metal and residual aluminum in treated water, which is linked to neurotoxicity [10,13,14]. Additionally, synthetic polymers have limited biodegradability, and polyacrylamide-based flocculants are of particular risk due to the potential presence of residual acrylamide monomers, which are recognized carcinogens [15,16,17]. These limitations have encouraged the development of more suitable pretreatment strategies [18,19].

Natural clarification agents include mineral materials (e.g., colloidal silica and bentonite) and biopolymers such as chitosan, cationic starches, tannin-based materials, and plant proteins. These materials promote the adsorption, aggregation, and sedimentation of suspended particles and are considered sustainable alternatives for wastewater pretreatment due to their biodegradability and low environmental impact [20].

For OMW applications aimed at phenolic recovery, clarification must remove suspended solids while minimizing phenolic losses. In this study, cationic starches were selected for the coagulation–flocculation step prior to adsorption on polymeric resins. Their positively charged functional groups neutralize the negatively charged particles in OMW, promoting the formation of larger flocs that are more easily removed by sedimentation [10,21,22,23,24].

Following clarification, the phenolic-enriched supernatant can be treated using nonionic macroporous resins, which selectively adsorb phenolic compounds while allowing most other OMW components to pass through. Adsorption is primarily driven by hydrophobic and π–π interactions between the aromatic structures of the phenolics and the resin matrix [25,26,27]. As these resins can be regenerated and reused, they represent an efficient approach for concentrating phenolics from OMW.

Our previous characterization of Dalmatian OMW showed high total phenolic content and strong antioxidant activity, with hydroxytyrosol as the dominant quantified phenolic compound [28]. Building on those findings, the novelty of this study lies in evaluating cationic starch clarification not as an endpoint wastewater treatment step, but as a pretreatment designed to preserve phenolics and improve subsequent resin-based recovery of hydroxytyrosol-rich, antioxidant-active fractions from Dalmatian OMW.

Accordingly, the present study aimed to develop and evaluate an integrated, multi-stage process combining cationic starch pretreatment and macroporous resin adsorption for the recovery of antioxidant-active phenolics from Dalmatian OMW. 

## 2. Materials and Methods

### 2.1. Samples

During the 2025 olive-processing season (October–November), 100 OMW subsamples (5 L each) were collected from olive mills located in Central Dalmatia, a region characterized by the cultivation of several olive cultivars. Samples were taken from wastewater discharge points immediately after horizontal or vertical centrifugation, depending on the mill configuration, and were combined to obtain a 500 L composite OMW sample used in the present study. Since the primary aim was to establish a proof-of-concept protocol for polyphenol extraction, cultivar-specific differences were not investigated. Instead, emphasis was placed on obtaining a sufficiently homogeneous composite sample from mill locations that had shown the highest phenolic concentrations in our previous study. A detailed description of the sampling locations, mill configurations, and collection procedure has been reported previously [28]. The subsamples were pooled and homogenized in a plastic barrel to obtain a composite sample, hereafter referred to as the original OMW. All measurements and determinations were made in duplicate.

**Figure 1:**
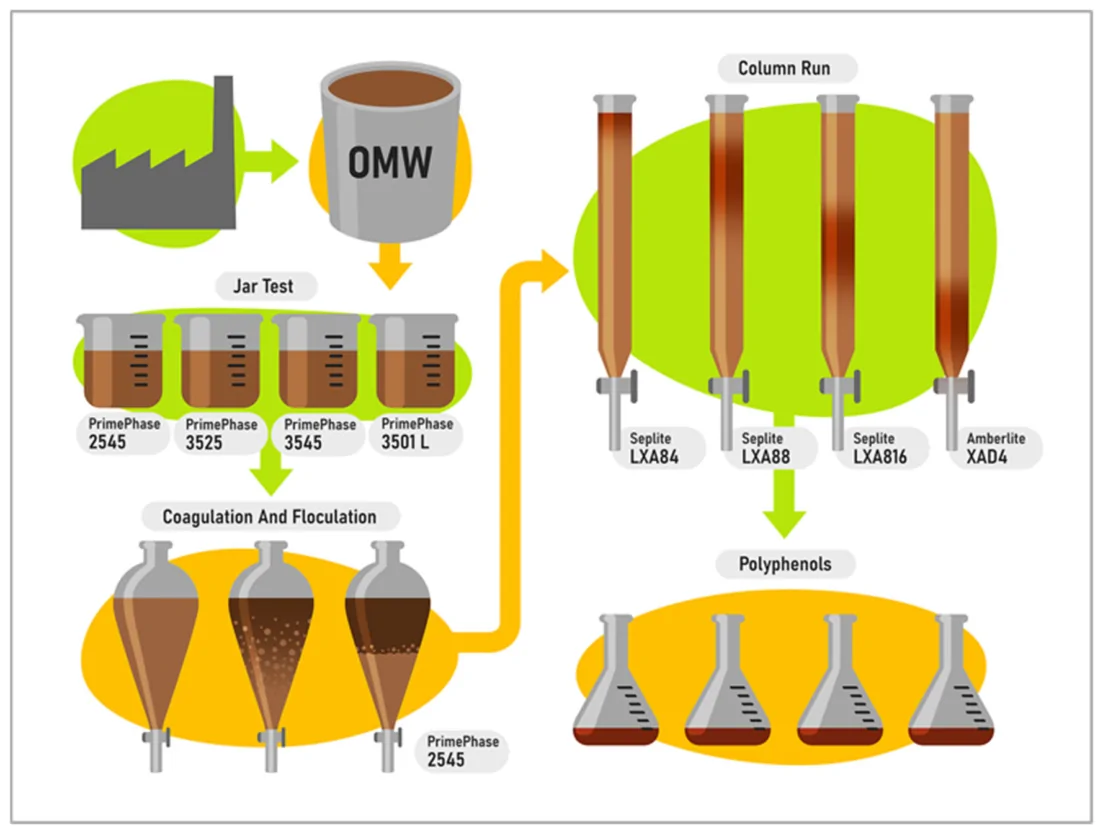
Experimental workflow for the recovery of phenolic compounds from olive mill wastewater.

The experimental workflow of the present study is summarized in Figure 1.

### 2.2. Characterization of OMW

The homogenized OMW sample was characterized using the following physicochemical parameters: pH value [29], electrical conductivity [30], color [31], odor [32], suspended solids [33], chemical oxygen demand (COD) [34], biological oxygen demand after 5 days (BOD_5_) [35], and total organic carbon (TOC [36]).

### 2.3. Total Phenolic Content (TPC)

Total phenolic content (TPC) was determined using the Folin–Ciocalteu spectrophotometric method with minor modifications [37]. The original OMW was centrifuged for 15 min at 5000× *g* (Tehtnica 4GT centrifuge; Iskra, Ljubljana, Slovenia), and sequentially filtered through a glass-fiber (1.2 μm pore size) and nylon (0.45 μm) syringe filters. Processed OMW samples were analyzed directly after appropriate clarification. A 0.1 mL aliquot was transferred to a 25 mL volumetric flask and diluted to 5 mL with ultrapure water (0.05 mS cm^−1^; Omnia Pure, Stakpure GmbH, Niederahr, Germany). Folin–Ciocalteu reagent (0.5 mL; Sigma-Aldrich, St. Louis, MO, USA) was added, followed after 3 min by 1 mL of 20% (*w*/*v*) Na_2_CO_3_ solution (Sigma-Aldrich, St. Louis, MO, USA). The volumetric flask was filled to the final volume (25 mL) with ultrapure water. Samples were kept in the dark for 60 min, and absorbance was measured at 760 nm using a UV-Vis spectrophotometer (Lambda 25, PerkinElmer, Shelton, CT, USA). The calibration curve was prepared with gallic acid standard solutions (Sigma-Aldrich, St. Louis, MO, USA) over the range 5 to 20 μg mL^−1^, yielding a linearity (R^2^) of 0.999.

### 2.4. HPLC Characterization of Phenolic Compounds

The phenolic profile was determined by High-Performance Liquid Chromatography (HPLC) directly in OMW samples without prior solvent extraction. The untreated homogenized OMW was prepared by centrifugation and filtration as described above. Chromatographic analyses followed COI/T.20/Doc. No 29/Rev.2/2022 [38] and were performed on a PerkinElmer Series 200 HPLC system (PerkinElmer, Waltham, MA, USA) equipped with a UV–VIS detector operating at 280 nm. Separation was achieved on an Ultra Aqueous C18 column (250 × 4.6 mm, 5 μm particle size; Restek, Bellefonte, PA, USA). 

The mobile phases were 0.2% phosphoric acid in water (A) and a methanol/acetonitrile mixture (1:1, *v*/*v*) (B). The gradient program used for elution started at 4% B, increased to 50% B over 40 min, to 60% over the next 5 min, and to 100% B between 45 and 60 min.

After maintaining isocratic conditions at 100% B for 8 min, the composition was returned to the initial 4% B between 68 and 70 min and maintained for an additional 10 min to re-equilibrate the column. The column temperature was 25 °C, the flow rate was 0.8 mL min^−1^, and the injection volume was 20 μL.

### 2.5. Determination of Antioxidant Capacity

The antioxidant capacity of the recovered fractions was evaluated using three complementary spectrophotometric assays based on distinct reaction mechanisms. DPPH and ORAC assays were used to assess antioxidant activity via hydrogen atom transfer (HAT), whereas the FRAP assay was used to determine the reducing potential of the samples via electron transfer (ET). Measurements were performed in 96-well microplates using a Sunrise microplate reader (Tecan, Männedorf, Switzerland). Analyses were carried out in triplicate, and the results were expressed as mean values ± standard deviation using Trolox as the reference standard (mM Trolox equivalents, mM TE).

#### 2.5.1. DPPH Radical Scavenging Assay

DPPH radical-scavenging activity was determined as described previously [28]. A DPPH stock solution (4 mg/100 mL ethanol) was diluted to obtain an absorbance of 1.20 ± 0.02 at 517 nm. For each measurement, 290 µL of working DPPH solution was added to a microplate well and the initial absorbance was then recorded. Subsequently, 10 µL of appropriately diluted extract (1:20) was added to the reaction mixture. Following a 60 min incubation period, the decrease in absorbance was measured and used as an indicator of radical-scavenging capacity.

#### 2.5.2. Ferric Reducing Antioxidant Power (FRAP) Assay

The reducing power of the extracts was assessed using the FRAP assay as previously described by Knezović et al. [28]. Briefly, 300 µL of freshly prepared FRAP reagent was added to each well, and the initial absorbance was measured at 592 nm. Thereafter, 10 µL of sample was introduced into the reaction system. The increase in absorbance resulting from the reduction in the ferric–tripyridyltriazine complex to its ferrous form was recorded after 4 min and used to estimate the reducing capacity of the extracts.

#### 2.5.3. Oxygen Radical Absorbance Capacity (ORAC) Assay

The antioxidant potential was further evaluated using the ORAC assay, which is based on the inhibition of peroxyl radical-induced oxidation of a fluorescent probe [28]. The reaction mixture contained 150 µL fluorescein solution and 25 µL of sample, Trolox standard, or the blank solution, followed by incubation at 37 °C for 30 min. Oxidative degradation was initiated by adding 25 µL AAPH solution as a peroxyl radical generator. Fluorescence was monitored kinetically over 90 min, and antioxidant capacity was calculated from the area under the fluorescence decay curve relative to the Trolox.

### 2.6. Experimental Design and Sample Preparation 

#### 2.6.1. Coagulation–Flocculation Screening by Modified Jar Test

Coagulation–flocculation was applied to the homogenized OMW to remove suspended solids, turbidity, and sediment while minimizing the loss of dissolved phenolic compounds. Four cationic starches were tested: PrimePHASE 2545, PrimePHASE 3525, PrimePHASE 3545, and PrimePHASE 3501L (kindly provided by Chemigate Oy, Mietoinen Finland). Their main characteristics are presented in Table 1.

To determine the optimal concentrations of active substances for impurity removal, a modified jar-test was performed. For each cationic starch, eight 500 mL OMW samples were treated with equal volumes of differently concentrated starch stock solutions to achieve active-substance doses ranging from 10 to 1000 mg L^−1^ (Table S1). Because a conventional multi-position jar-test apparatus was not available, experiments were conducted sequentially on individual samples rather than simultaneously. After the addition of starch, each sample was mixed using a UM-405 stirrer (Tehtnica, Železniki, Slovenia): rapid mixing at 350 rpm for 1 min to disperse the coagulant, followed by slow mixing at 60 rpm for 15 min to promote floc formation and subsequent settling. The same mixing times, stirring rates, reagent dosages, and settling conditions were used across treatments to ensure comparability of the results.

#### 2.6.2. Coagulation and Flocculation—OMW Sample 

Based on the jar-test results, the remaining homogenized OMW was clarified using the best-performing cationic starch at the concentration which obtained optimum results. Sedimentation was carried out in 2 L separatory funnels, with 200 mL of cationic starch solution added, achieving a concentration of 1000 mg active substance L^−1^ in OMW. Immediately after addition, the sample was mixed with a stirrer for 1 min at 350 rpm, and then for 15 min at 60 rpm. The sample was left for 12 h. Fine sediment was removed through the funnel valve, and the clarified supernatant was filtered sequentially through 8–12 μm and 2–3 μm filter papers (Fioroni Filters, Ingré, France).

#### 2.6.3. Isolation of Polyphenols Using Adsorption Resins

The filtered supernatant was passed through an adsorption resin column to isolate and concentrate the polyphenolic fraction. Four resins were evaluated: Seplite LXA84, Seplite LXA88, Seplite LXA816 (kindly provided by Sunresin New Materials Co., Ltd., Xi’an, Shaanxi, China) and Amberlite XAD4 (Sigma Aldrich, Saint-Quentin-Fallavier, France). Their reported characteristics are summarized in Table 2.

Glass columns equipped with sintered plates (5 cm in diameter and 50 cm in height, Deotto Lab, Zagreb, Croatia) were used for column experiments to extract polyphenols. Before use, resins were activated according to the manufacturer’s protocols (Table 3), as this was expected to provide the most appropriate working state for the respective material [39,40]. In all experiments, the resin bed height was adjusted to 23 cm, corresponding to a bed volume (BV) of approximately 450 mL.

After activation, 1 L of clarified OMW was acidified with hydrochloric acid (to pH = 2.5) and loaded onto each resin column, and phenolics were eluted with 75% ethanol. Each column run was monitored by collecting 100 mL fractions. TPC was measured in each fraction to evaluate adsorption, breakthrough, and desorption. Fractions with the highest measured TPC were selected for antioxidant-activity testing and HPLC phenolic profiling. Each resin was evaluated in one independent column run as part of a preliminary screening experiment. Duplicate TPC and HPLC measurements and triplicate antioxidant activity measurements represented analytical replicates and were used to assess analytical precision rather than the reproducibility of the complete adsorption process. To assess whether acidification affected the overall phenolic content, TPC was measured before and after adjustment to a pH of approximately 2.5. No measurable difference was observed, indicating that acidification did not cause a detectable loss of total Folin-reactive phenolic material under the applied experimental conditions.

### 2.7. Data Presentation and Recovery Calculations

Data are presented descriptively. The collected OMW subsamples were pooled into a single composite OMW matrix before analysis; therefore, the study was designed as a process-screening evaluation rather than as an assessment of biological, seasonal, cultivar-related, or mill-to-mill variability. Measurements and determinations were performed in duplicate unless otherwise stated. Antioxidant activity assays were performed in triplicate and are presented as mean ± standard deviation. For the jar-test screening, mean and standard deviation values across the eight tested cationic-starch concentrations were used only as descriptive summaries of the screening range and were not treated as independent experimental replicates. Each resin was evaluated in a single column run, the results are presented descriptively, and differences among resins should be interpreted as preliminary observations under the applied experimental conditions. No inferential statistical comparisons between treatments or resins were performed. Phenolic retention, recovery, breakthrough, and mass-balance closure were calculated from measured concentrations and corresponding sample or fraction volumes and expressed as percentages relative to the relevant initial, loaded, or recovered phenolic amount. For HPLC-based phenolic profiling, concentrations of individual phenolic compounds were converted into absolute masses to account for the different volumes of the loaded sample and the collected ethanol fractions. The original OMW value represents the mass of quantified phenolics present in the 1 L sample loaded onto the column. In contrast, resin eluate values represent the mass of quantified phenolics present in the selected 100 mL ethanol desorption fraction. This approach was used because resin adsorption concentrates phenolics from the loaded OMW into a smaller elution volume. Therefore, comparison based solely on concentration would overestimate differences resulting from volume reduction.

## 3. Results and Discussion

### 3.1. Characteristics of the OMW Sample

The homogenized OMW had a pH of 4.27 and a very high odor intensity (10,000 TON) (Threshold Odor Number (TON)), while the color could not be determined because of the sample density. The original sample also contained high suspended solids (5740 mg L^−1^), COD (111,300 mg O_2_ L^−1^), BOD_5_ (56,307 mg O_2_ L^−1^), and TOC (30,243 mg C L^−1^). The total phenolic content, calculated from the gallic acid calibration curve, was 10.65 μg GAE mL^−1^ (gallic acid equivalents (GAE)) in the diluted analytical solution. After correction for the dilution factor, the total phenolic content of the original OMW sample was 2663 mg GAE L^−1^ OMW.

These values confirm that untreated OMW represents a major environmental burden due to its high organic load, suspended solids, and phenolic content. Uncontrolled discharge may deplete dissolved oxygen in receiving waters, disrupt aquatic ecosystems, and alter soil pH, microbial activity, and plant growth. Phenolic compounds are of particular interest because they contribute both to the toxicity of OMW and to its potential as a source of natural antioxidants. 

The purpose of the cationic-starch pretreatment was therefore twofold: to reduce the suspended and organic matter sufficiently for subsequent column operation, and to retain as much of the dissolved phenolic fraction as possible for recovery in the adsorption step. 

### 3.2. Jar Test

A modified jar test was performed to identify the most suitable active-substance concentration for each cationic starch, as described in Section 2.6.1.

**Figure 2:**
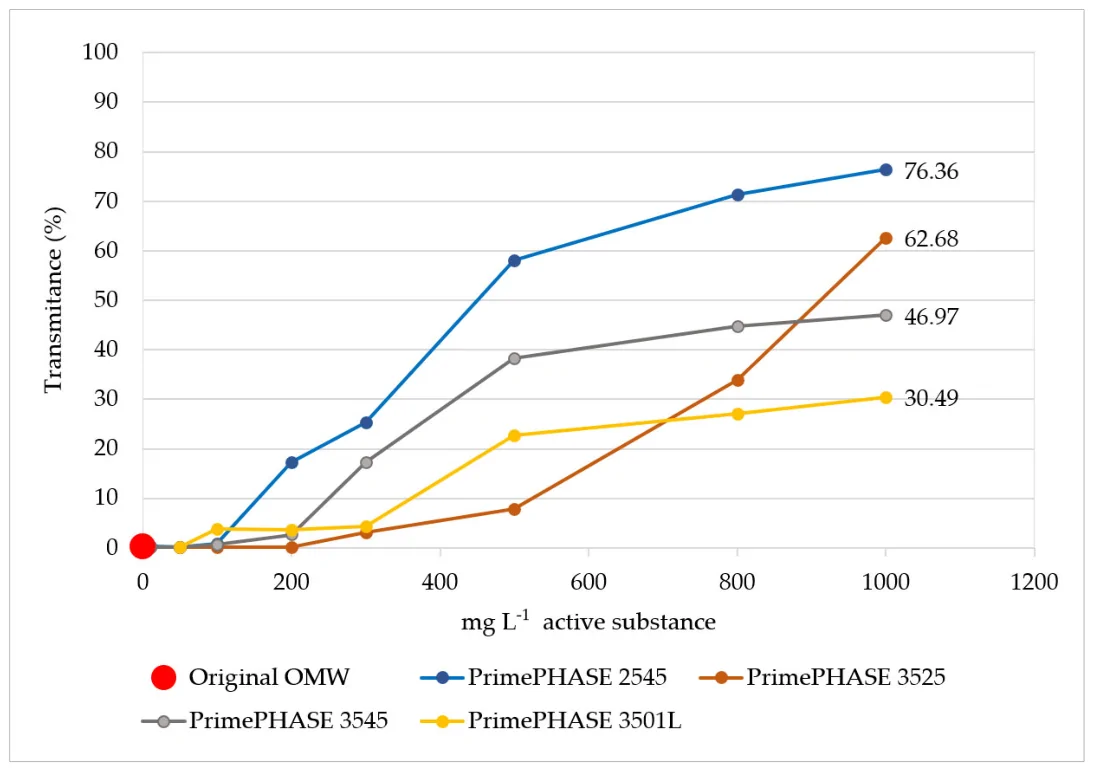
Transmittance at 700 nm (T700) of OMW samples after treatment with cationic starches; PrimePHASE 2545, PrimePHASE 3525, PrimePHASE 3545, and PrimePHASE 3501L. Higher values indicate greater clarification. Red dot represents original OMW sample without starch addition.

For each starch, eight active-substance concentrations were tested. After sedimentation, transmittance at 700 nm (T_700_) was measured as a relative indicator of clarity; higher transmittance indicates lower turbidity [41,42]. The original OMW was used as a control sample and subjected to the same experimental conditions of mixing and sedimentation as samples to which different concentrations of starch were added. The results are shown in Figure 2.

**Figure 3:**
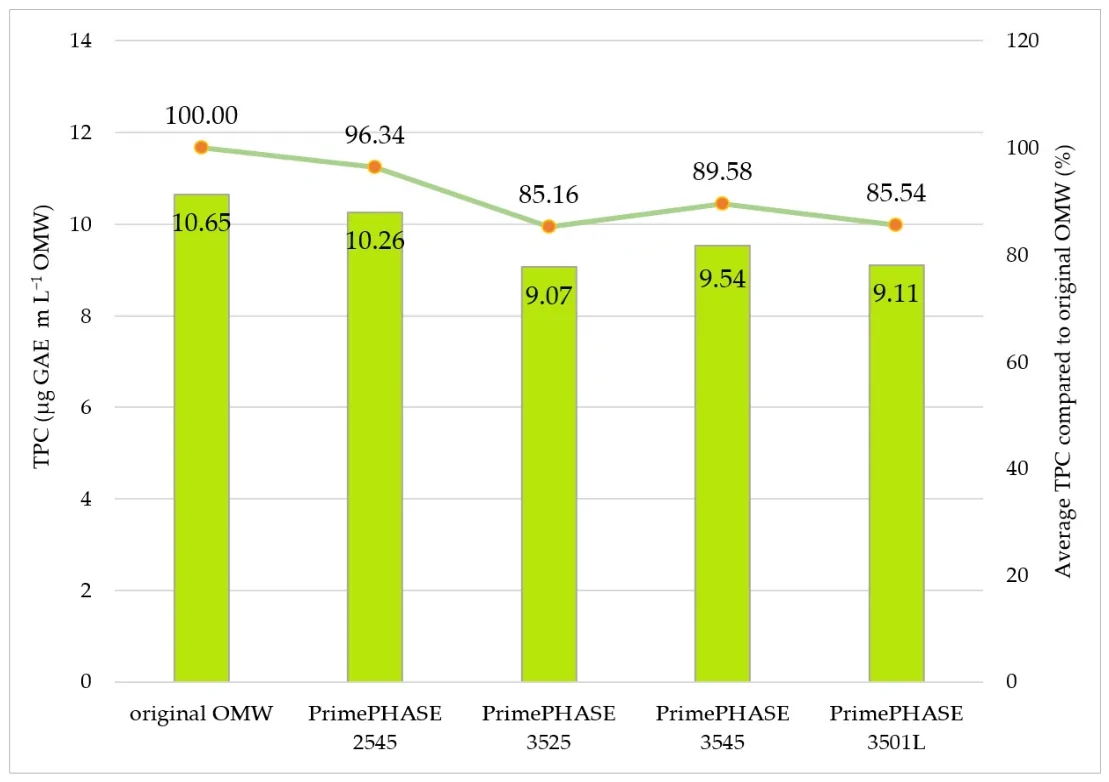
Total phenolic content of OMW samples during the jar test optimization. Mean TPC values with standard deviation are shown on the primary y-axis, and values relative to the original OMW are shown on the secondary y-axis. Bars represent mean values ± SD.

The main objective of this study was the extraction of polyphenols from OMW. Therefore, in addition to monitoring the clarification of the samples, TPC concentrations were measured in all samples during the jar test, in order to check whether there were any losses in polyphenol content during the precipitation. As shown in Figure 3 and Table S1, all tested cationic starches resulted in some loss of total phenolic content (TPC) during precipitation. TPC retention was highest for PrimePHASE 2545, followed by PrimePHASE 3545, whereas PrimePHASE 3525 and PrimePHASE 3501L showed lower and comparable retention values.

The physicochemical and organic characteristics of OMW after treatment with four cationic starches at 1000 mg active substance L^−1^ are compared with those of the original OMW in Table 4.

Increasing the cationic starch dose generally increased transmittance, indicating more intensive coagulation–flocculation and improved OMW clarification (Figure 2). At 1000 mg L^−1^ active substance, PrimePHASE 2545 provided the most favorable balance, achieving the highest transmittance (76.36%) while retaining 96.34% of the initial TPC (Figure 3, Table S1). PrimePHASE 3525 achieved relatively high transmittance (62.68%) but showed lower TPC retention (85.16%). PrimePHASE 3545 provided moderate clarification (46.97%) with comparatively better TPC retention (89.58%), whereas PrimePHASE 3501L showed both the lowest transmittance (30.49%) and relatively low TPC retention (85.54%). These results indicate that improved clarification may be accompanied by some phenolic loss, although the extent of this trade-off depended on the starch formulation.

The superior performance of PrimePHASE 2545 may be related to its balance between medium cationic charge density and high chain length. This structure likely provided sufficient charge neutralization to destabilize negatively charged colloids, while long polymer chains promoted interparticle bridging and formation of larger, settleable flocs. In contrast, starches with a higher cationic charge could increase the risk of overdosing, charge reversal, or non-selective removal of dissolved organic matter at the tested dose. PrimePHASE 3501L, despite its high active-solids content and high cationic charge, has a very low chain length, which may limit bridging flocculation. Therefore, PrimePHASE 2545 provided the most favorable compromise between matrix clarification and phenolic preservation [43,44,45].

Accordingly, PrimePHASE 2545 at 1000 mg of active substance L^−1^ was selected for pretreatment of the remaining homogenized OMW before adsorption resin experiments.

### 3.3. Extraction of Polyphenols on Adsorption Resins

Four macroporous adsorption resins were compared for their ability to retain and desorb OMW phenolics: Seplite LXA84, Seplite LXA88, Seplite LXA816, and Amberlite XAD4. Since OMW is acidic (pH approximately 4), many phenolic compounds are expected to be predominantly in neutral or protonated forms. Under these conditions, ion exchange is unlikely to be the dominant retention mechanism; instead, hydrophobic interactions, hydrogen bonding, and π-π interactions between phenolic aromatic structures and the resin surface are expected to drive adsorption [46]. 

The resins were activated as described in Section 2.6.3. The OMW sample clarified with PrimePHASE 2545 was acidified with hydrochloric acid (to pH ≈ 2.5) and loaded onto each column at a flow rate of 0.5^−1^ BV/hour. Phenolics were desorbed using 75% ethanol. Sequential fractions were collected during loading, desorption, and washing, and phenolic concentration was measured to monitor breakthrough, desorption, and overall resin performance. The distribution of total phenolics among process fractions is presented in Table 5, which summarizes the descriptive phenolic mass distribution obtained from a single screening-level column run for each resin. Therefore, the observed differences should be interpreted as preliminary trends under the applied experimental conditions.

Values represent the percentage distribution of phenolic mass among the collected column-process fractions relative to the initial phenolic mass present in the clarified OMW loaded onto the column. For each 100 mL fraction, absolute phenolic mass was calculated by multiplying the measured TPC concentration by the corresponding fraction volume. The masses of individual fractions were then summed according to the process stage. Phenolics detected during sample loading were classified as breakthrough, whereas phenolics recovered in the ethanol fractions represented the target desorption recovery. Mass-balance closure was calculated as the sum of phenolic masses quantified in the initial aqueous effluent, loading effluent, ethanol eluate, and final aqueous wash. The difference from 100% was considered the unaccounted phenolic fraction. The unaccounted phenolic fraction was 23.12% for LXA84, 46.70% for LXA816, and 38.71% for XAD4. 

The high mass-balance closure observed for LXA88 (96.95%) did not reflect favorable adsorption performance. On the contrary, 61.00% of the initial phenolic load was detected in the loading effluent, indicating extensive breakthrough and poor phenolic retention by this resin. Its high closure therefore resulted mainly from the analytical recovery of non-adsorbed phenolics in the loading fractions. LXA84 showed an intermediate breakthrough of 14.92% but the highest ethanol-phase recovery of 60.53%, providing the most favorable balance between phenolic retention during loading and recovery in the technologically relevant ethanol fraction. In contrast, LXA816 and XAD4 showed very limited breakthrough, at 2.08% and 3.28%, respectively, indicating substantially greater retention during loading. However, their lower mass-balance closure, together with ethanol recoveries of 50.89% and 56.96%, suggests that part of the adsorbed phenolic material was not desorbed under the applied conditions and may have remained associated with the resin bed. Among the tested resins, LXA84 showed the most favorable overall performance, followed by Amberlite XAD4; however, independent replicate column experiments are required to confirm this observation. Although LXA84 and XAD4 belong to the same general class of nonionic, hydrophobic aromatic adsorbents, their similar styrene–DVB chemistry and nominal surface areas do not imply an identical porous structure. The resins may differ in pore-size distribution, accessible pore volume, degree of crosslinking, swelling behavior, particle morphology, and internal diffusion characteristics. However, these influences were not experimentally determined in this study.

**Figure 4:**
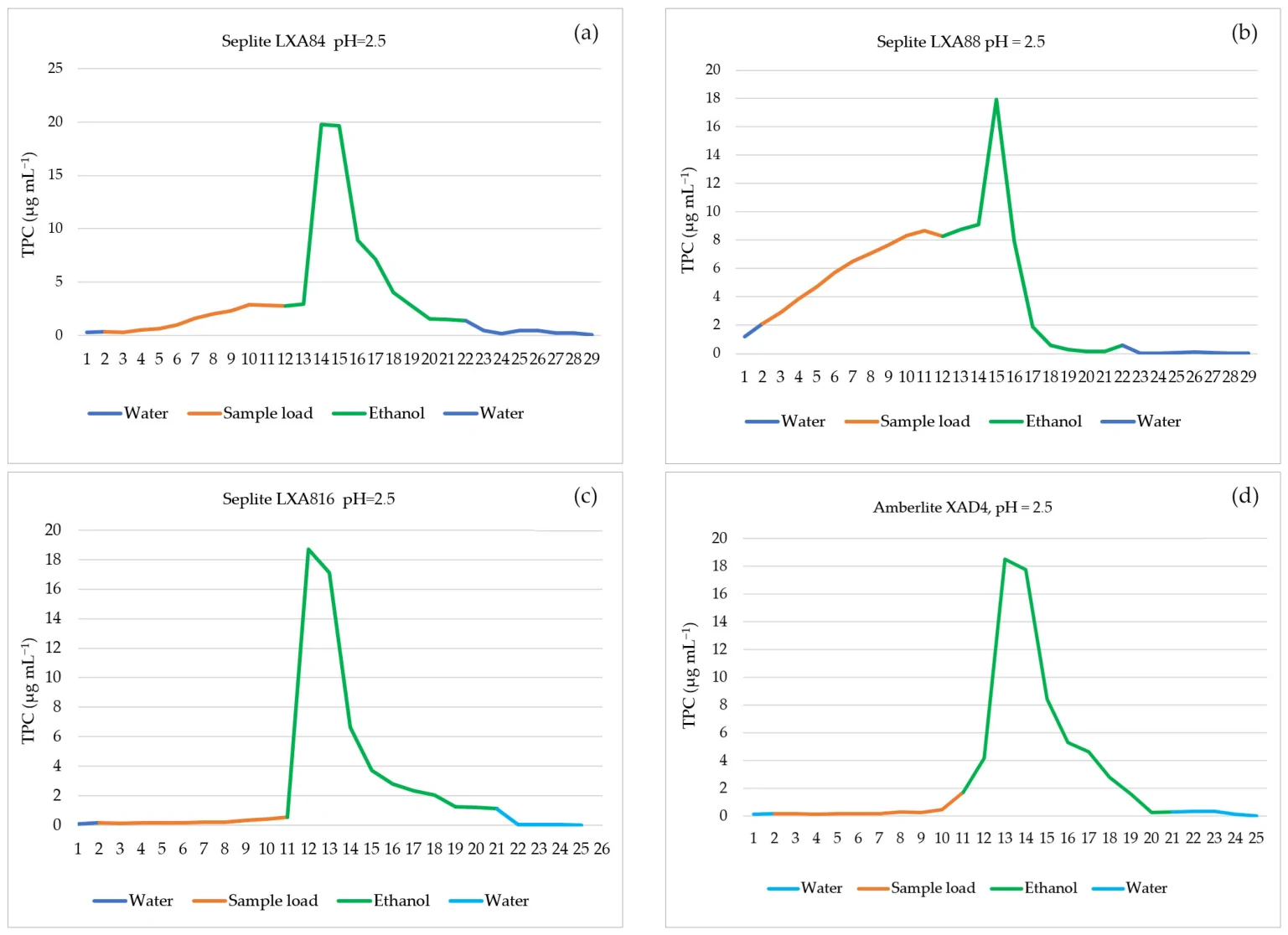
Elution profiles of total phenolics during the column runs with (**a**) Seplite LXA84, (**b**) Seplite LXA88, (**c**) Seplite LXA816, and (**d**) Amberlite XAD4. Numbers on the x-axis represent sequential 100 mL fractions collected during water wash, sample loading, ethanol desorption, and final water wash.

Figure 4 presents breakthrough and elution profiles of total phenolics during column runs with Seplite LXA84, Seplite LXA88, Seplite LXA816, and Amberlite XAD4. The curves show phenolic distribution across the main process phases: initial water wash, sample loading, ethanol desorption, and the final water wash.

The elution profiles revealed marked differences in adsorption and desorption behavior among the tested resins. Seplite LXA88 showed the highest mass-balance closure (96.95%; Table 5) but also pronounced breakthrough during sample loading (Figure 4b), indicating weak retention of phenolics under the applied conditions. Seplite LXA816 and Amberlite XAD4 showed minimal breakthrough, suggesting efficient adsorption. However, the low ethanol recovery and poor mass-balance closure observed for LXA816 indicate incomplete desorption, probably due to strong resin-phenolic interactions. Overall, in this preliminary study, Seplite LXA84 and Amberlite XAD4 exhibited the most favorable profiles, combining limited breakthrough with pronounced ethanol elution peaks.

### 3.4. Antioxidant Activity of Recovered Phenolic Fractions

Antioxidant activity was measured to evaluate the functional potential of the recovered phenolic fractions, because TPC alone does not fully describe antioxidant effectiveness. Antioxidant capacity depends on phenolic concentration, chemical structure, redox properties, and possible synergistic interactions with other extract constituents. Therefore, the use of complementary antioxidant assays provided additional information on the ability of the fractions to neutralize free radicals, reduce oxidizing agents, and inhibit oxidative processes.

**Figure 5:**
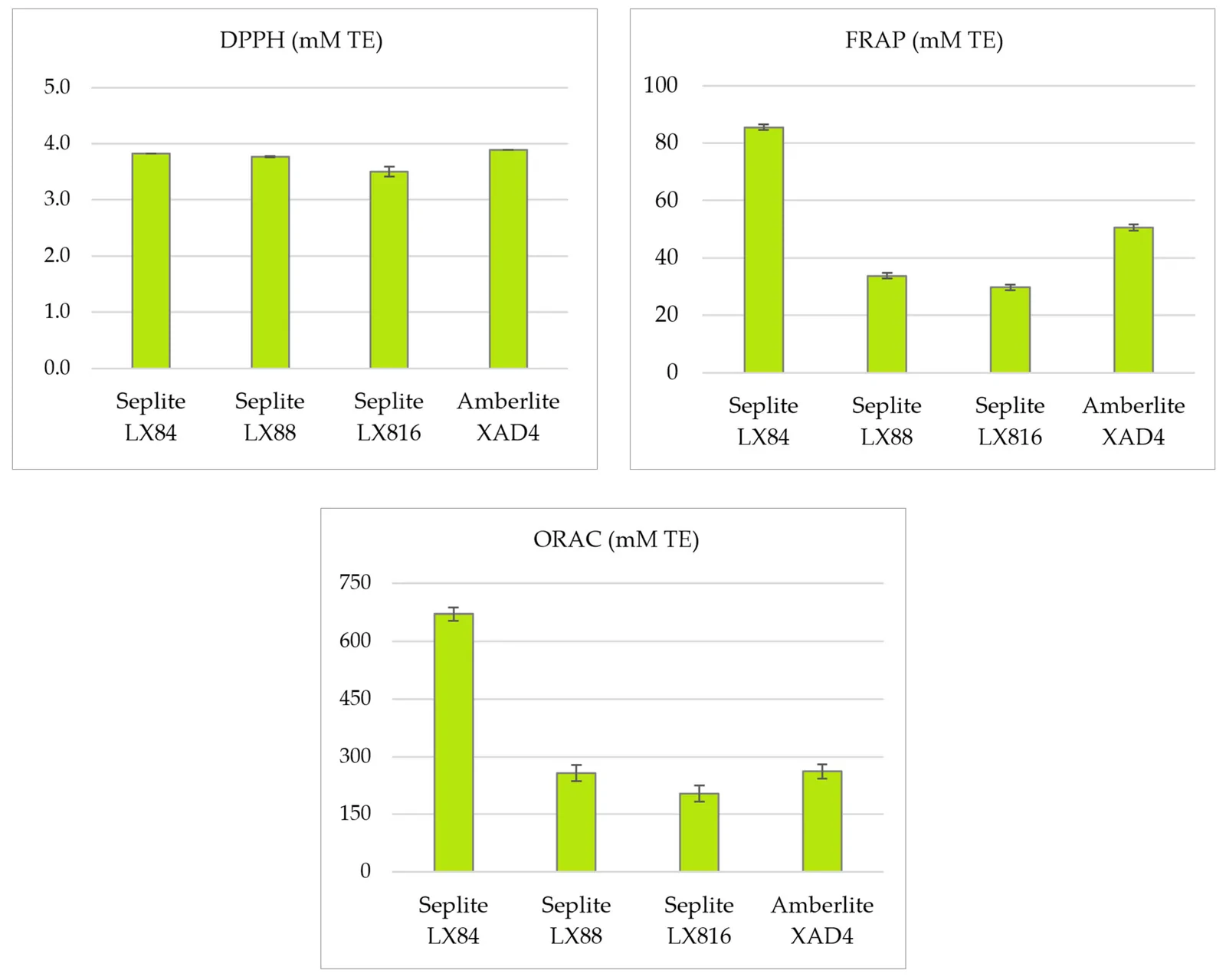
Antioxidant capacity of ethanol fractions obtained from the four adsorbent resins, measured using DPPH, FRAP, and ORAC inhibition assays. Values on the y-axis represent the antioxidant activity expressed as mM Trolox equivalents (mM TE).

Antioxidant activity was assessed using three complementary assays (FRAP, DPPH, and ORAC), which revealed differences between fractions using different adsorbent resins (Figure 5).

Among the tested resins, the ethanol fraction obtained with Seplite LXA84 showed the strongest overall antioxidant activity, exhibiting the highest FRAP (85.51 ± 0.75 mM TE) and ORAC values (670.47 ± 16.83 mM TE) together with high DPPH radical-scavenging activity (3.83 ± 0.00 mM TE) (Table S2). This indicates strong reducing and peroxyl-radical-scavenging properties. Amberlite XAD4 also produced an active fraction, with the highest DPPH value (3.89 ± 0.00 mM TE) and high FRAP activity (50.56 ± 0.71 mM TE), although its ORAC value (261.93 ± 18.72 mM TE) was considerably lower than that of Seplite LXA84. Seplite LXA88 and Seplite LXA816 fractions exhibited comparatively lower antioxidant activity, particularly in the FRAP and ORAC assays, despite their relatively high DPPH values (3.77 ± 0.01 and 3.50 ± 0.09 mM TE, respectively). The comparatively lower activity of the Seplite LXA816 eluate suggests that some antioxidant phenolics remained strongly retained on the resin or were incompletely desorbed under the applied elution conditions. The differences observed among the assays likely reflect both the phenolic composition of the resin fractions and the assay-specific reaction mechanisms and conditions [47]. The higher FRAP and ORAC values of Seplite LXA84 are consistent with its greater recovery of a hydroxytyrosol-rich phenolic fraction, supporting strong reducing and peroxyl radical-scavenging activity [48,49]. Amberlite XAD4 showed only a slightly higher value in the DPPH assay, which may reflect assay-specific differences in the reactivity of its phenolic compounds under ethanolic conditions [47,50]. Accordingly, this marginal difference should be interpreted as an assay-dependent response rather than as evidence of greater overall antioxidant capacity [51].

### 3.5. HPLC Profile of Recovered Phenolic Fractions

**Figure 6:**
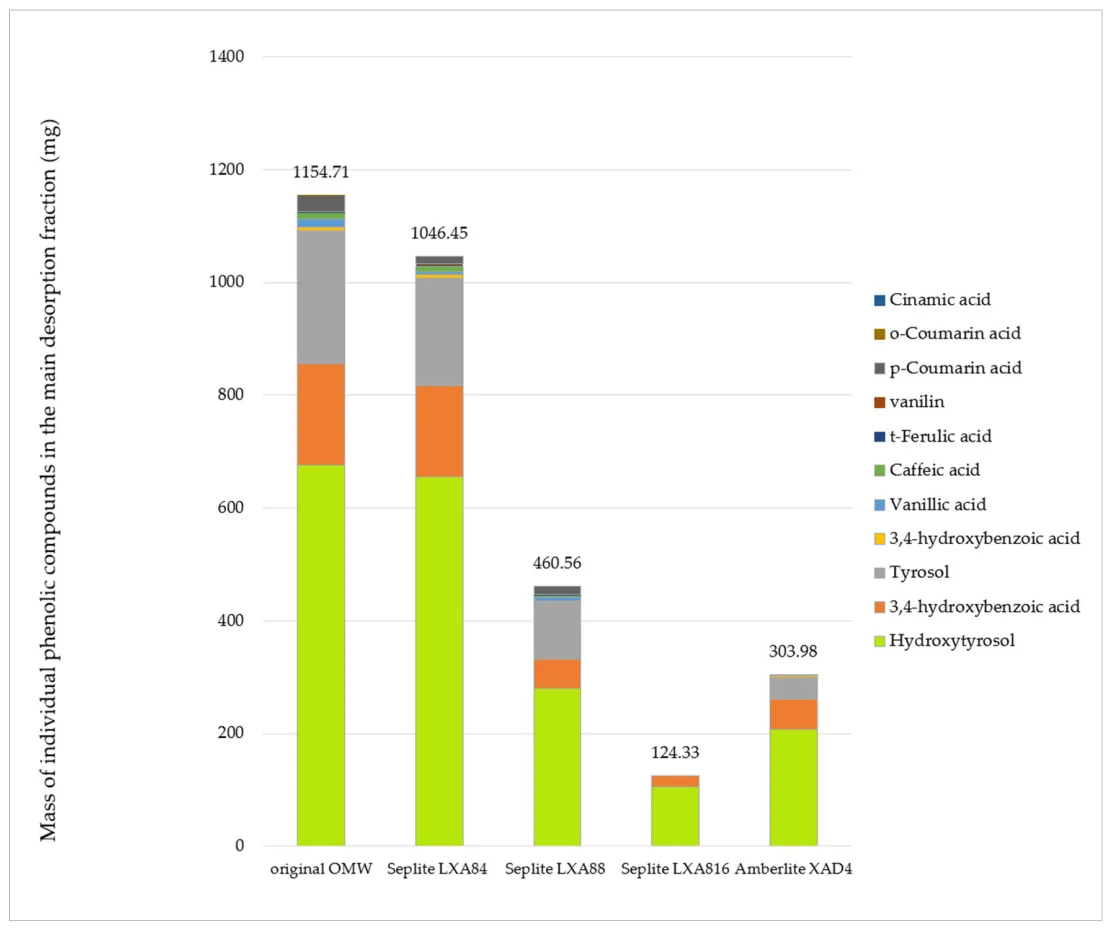
Phenolic profile of the original OMW and selected 100 mL desorption fractions obtained using different adsorbent resins. Values on the y-axis are expressed as absolute masses of quantified phenolic compounds. For the original OMW, masses were calculated for the 1 L sample loaded onto the column; for resin eluates, masses were calculated for the selected 100 mL desorption fraction.

The HPLC-determined concentrations of individual phenolic compounds in the original OMW and in the selected 100 mL ethanol desorption fractions are presented in Table S3. This fraction was selected because it contained the highest TPC concentration and was therefore considered the most suitable candidate for subsequent concentration and potential valorization. Because the adsorption step concentrated phenolic compounds from 1 L of clarified OMW into selected ethanol fractions, the concentrations measured in the eluates were higher, as expected, than those in the original OMW. Therefore, for graphical comparison of compound-specific recovery, HPLC concentrations were additionally converted into absolute masses and presented in Figure 6. This allowed the compound-specific recovery pattern to be evaluated independently of the concentration effect caused by volume reduction during elution. However, the summed HPLC mass in these selected fractions does not represent process recovery; rather, it represents a comparison of phenolic profiles after desorption from the resin and the original OMW sample.

Hydroxytyrosol was the dominant phenolic compound in all samples, suggesting that the recovered fractions were enriched mainly in low-molecular-weight phenolics with high antioxidant relevance. Tyrosol and 3,4-hydroxybenzoic acid were also present in notable amounts, whereas vanillin, vanillic acid, caffeic acid, ferulic acid, cinnamic acid and coumaric acid derivatives were detected at lower levels. Among the tested resins, Seplite LXA84 showed the highest efficiency for retaining and desorbing the identified phenolics. The quantified phenolic mass in the main Seplite LXA84 concentrated desorption fraction (100 mL) was 1046.45 mg, close to the value determined for the original OMW sample (1154.71 mg). This suggests that Seplite LXA84 provided the closest recovery of the original phenolic profile, particularly for hydroxytyrosol. Seplite LXA88 retained the main phenolic compounds to some extent, but its overall performance was limited by losses during loading (Figure 4b), indicating early breakthrough and insufficient retention. The lowest amounts of identified compounds were observed in the desorption fractions obtained with Seplite LXA816 and Amberlite XAD4. For these resins, the elution profiles suggest that strong binding and incomplete desorption, rather than poor adsorption alone, may explain the lower recovered amounts. Further optimization of ethanol concentration, eluent volume, flow rate, and pH is therefore needed to improve total phenolic recovery.

The phenolic profile was consistent with the antioxidant-activity results. Fractions enriched in hydroxytyrosol and other phenolics generally showed higher antioxidant activity, which agrees with the strong reducing and radical-scavenging properties of hydroxytyrosol. The higher antioxidant activity observed for the Seplite LXA84 eluate can therefore be attributed to the greater total amount of recovered phenolics and the preservation of a hydroxytyrosol-dominated profile.

### 3.6. Study Strengths and Limitations

The main strength of this study is its integrated process-oriented design, which combined OMW clarification with subsequent adsorption resin purification rather than evaluating these steps separately. This approach allowed the preservation of phenolic compounds during pretreatment to be assessed together with their downstream recovery and antioxidant activity. Another strength is the comparative screening of several commercial cationic starches and adsorption resins under the same experimental framework, enabling the selection of the most suitable clarification–adsorption combination. In addition, the recovered fractions were characterized using multiple analytical endpoints, including conventional wastewater parameters, TPC, HPLC phenolic profiling, and antioxidant capacity assessed by three assays. This combination provides a broader assessment of process performance than TPC recovery alone. This study also has several limitations. The first limitation was that the jar-test experiments were conducted sequentially on individual samples rather than simultaneously, due to the unavailability of a conventional multi-position jar-test apparatus. Although the same experimental conditions were applied throughout the study, possible temporal variations in OMW properties between sequential runs cannot be entirely ruled out and should be considered when interpreting the comparative results.

Second, the resin experiments were performed as preliminary screening-level trials; with one independent process run performed for each tested resin, so the observed differences among resins should be interpreted descriptively. Third, total phenolic content was determined using the Folin–Ciocalteu assay, which is not specific only for phenolic compounds and may respond to other reducing substances present in OMW. For this reason, TPC-based recovery estimates were interpreted together with HPLC phenolic profiling and antioxidant activity assays. Further studies using independently collected OMW batches, replicated column runs, optimized desorption conditions, and expanded compound-specific recovery calculations are needed before scale-up or application-specific conclusions can be made.

## 4. Conclusions

This study indicates that efficient recovery of phenolic compounds from OMW requires an integrated process combining matrix clarification with adsorption-based concentration. Because OMW contains suspended solids, colloids, dissolved organic matter, and oil droplets, pretreatment is essential to reduce matrix interference before column adsorption while retaining the target phenolic fraction.

Among the tested cationic starches, PrimePHASE 2545 at 1000 mg active substance L^−1^ provided the best balance between clarification efficiency and phenolic retention. In this preliminary adsorption screening, Seplite LXA84 produced the most favorable phenolic-rich ethanol fraction, with high recovery of hydroxytyrosol-dominated phenolics and the strongest overall antioxidant activity. Amberlite XAD4 also showed promising adsorption and desorption behavior, whereas Seplite LXA88 suffered from breakthrough and Seplite LXA816 from incomplete desorption under the applied conditions.

Overall, the findings support cationic starch clarification followed by macroporous resin adsorption as a promising valorization route for producing antioxidant-active phenolic fractions from OMW. Future work should further optimize the extraction process, and evaluate the chemical stability, safety, and application performance of the recovered extracts. 

## Figures and Tables

**Table 1:** Characteristics of cationic starches used in this study.

Cationic Starch	Active Solids (%)	Degree of Cationic Charge	Chain Length
PrimePHASE 2545	15.0 ± 1	medium *	high *
PrimePHASE 3525	20.0 ± 1	high *	medium *
PrimePHASE 3545	20.0 ± 1	high *	high *
PrimePHASE 3501L	24.0 ± 1	high *	very low *

* data provided by the manufacturer.

**Table 2:** Characteristics of adsorbent resins used in this study.

Resin	Polymer Matrix	Ionic Character	Porosity Type/Pore-Size Information	Surface Area (m^2^/g)
Seplite LXA84	styrene/DVB	hydrophobic/aromatic	macroporous; small-pore class *	≥700
Seplite LXA88	styrene/DVBR	hydrophobic/aromatic	macroporous; special matrix structure *	NR *
Seplite LXA816	styrene/DVB	hydrophobic/aromatic	macroporous; medium-pore class *	≥900
Amberlite XAD4	styrene/DVB	hydrophobic/aromatic	macroporous; mean pore diameter ≈100 Å	750

DVB—divinylbenzene; NR = not reported; * For the Seplite resins, qualitative pore-size classes are reported according to the manufacturer because numerical mean pore diameters were not available.

**Table 3:** Activation protocol for adsorbent resins used in this study.

Adsorbent Resin	Activation Steps	
Seplite LXA84, Seplite LXA88, Seplite LXA816	1.	Soak the resin in deionized water overnight
	2.	Wash with 4% NaOH (1–2 BV/h)
	3.	Rinse with deionized water until pH < 10
	4.	Wash with 4% HCl (1–2 BV/h)
	5.	Rinse with deionized water until pH > 4
	6.	Soak resin in >95% ethanol for 2–4 h
	7.	Rinse with deionized water until ethanol content is <1%
Amberlite XAD4	1.	Soak the resin in methanol for 15 min
	2.	Decant methanol and add deionized water
	3.	Soak the resin in deionized water for 10 min

BV—bed volume, NaOH—Sodium hydroxide, HCl—Hydrochloric acid.

**Table 4:** The physicochemical and organic characteristics of wastewater after treatment with four types of cationic starches compared to the original OMW.

Parameter	Original OMW	PrimePHASE 2545	PrimePHASE 3525	PrimePHASE 3545	PrimePHASE 3501L
pH (25 °C)	4.27	4.37	4.36	4.35	4.35
Electrical conductivity (mS cm^−1^, 25 °C)	10.49	7.68	7.67	7.76	7.66
Color (mg L^−1^ Pt Co)	ND	4857	5216	4035	5138
Suspended solids (mg L^−1^)	5740	202	225	279	376
COD (mgO_2_ L^−1^)	111,300	21,000	18,600	19,400	23,700
BOD_5_ (mgO_2_ L^−1^)	56,307	11,245	10,763	9050	11,500
TOC (mg C L^−1^)	30,243	9300	10,200	10,150	9871

ND—not determined because of high sample density.

**Table 5:** Mass distribution of total phenolics among process fractions during the column runs, expressed as percentage of the initial phenolic load.

Process Fractions	Seplite LXA84	Seplite LXA88	Seplite LXA816	Amberlite XAD4
Aqueous fraction loss	0.58	2.93	0.24	0.28
Breakthrough during loading	14.92	61.00	2.08	3.28
Ethanol elution recovery	60.53	32.85	50.89	56.96
Final aqueous wash	0.84	0.17	0.09	0.77
Total accounted phenolic fraction	76.88	96.95	53.30	61.29

## Data Availability

The data presented in this study are available upon request from the corresponding author.

## Supplementary Materials

The following supporting information can be downloaded at: https://www.mdpi.com/article/10.3390/antiox15080946/s1, Table S1: Total phenolic content (TPC) in the samples treated during the jar test. Table S2: Antioxidant capacity of ethanol fraction (the concentration peak of desorption) measured by FRAP, ORAC, and DPPH assays. Table S3: HPLC-determined concentrations of the main polyphenolic compounds in the original OMW and in selected 100 mL ethanolic desorption fractions collected at the peak of elution from adsorbent resins.

## Abbreviations

The following abbreviations are used in this manuscript:

AAPH	2,2′-Azobis(2-amidinopropane) dihydrochloride
TON	Threshold Odor Number
BOD_5_	Biochemical oxygen demand after 5 days
BV	Bed volume
COD	Chemical oxygen demand
DPPH	2,2-Diphenyl-1-picrylhydrazyl
FRAP	Ferric reducing antioxidant power
GAE	Gallic acid equivalents
HPLC	High-performance liquid chromatography
OMW	Olive mill wastewater
ORAC	Oxygen radical absorbance capacity
TE	Trolox equivalents
TOC	Total organic carbon
TPC	Total phenolic content
UV-Vis	Ultraviolet-visible
