# Supplementary material for: An Integrated Screening Study of Polyphenol Recovery from Olive Mill Wastewater Using Cationic Starch-Assisted Clarification and Adsorption Resin Purification
Source: Antioxidants (Basel). 2026 Jul 29;15(8):946. doi: 10.3390/antiox15080946 (PMC13509548; doi:10.3390/antiox15080946)
Supplement: Supplementary file 1 [file antioxidants-15-00946-s001.zip › antioxidants-4438699-supplementary-revised-4-updated.pdf]

antioxidants

MDPI

Table S1. Total phenolic content (TPC) in the samples treated during the jar test.

Samples 1–8 correspond to treatments with different concentrations of cationic starches used as flocculants (10-1000 mg L^{-1}). Data are presented as mean values of measurements (each sample was measured in duplicate).

Jar test samples	Concentration of the cationic starch (mg L^{-1} OMW)	Total phenolic content (TPC), µg mL^{-1}				
		Original OMW	PrimePHASE 2545	PrimePHASE 3525	PrimePHASE 3545	PrimePHASE 3501L
		10,65				
1	10		9.40	9.64	9.31	8.90
2	50		9.33	9.13	9.27	8.74
3	100		9.60	9.20	9.38	9.16
4	200		9.90	8.86	9.03	9.22
5	300		10.25	9.55	9.70	9.14
6	500		10.49	9.69	9.88	9.09
7	800		9.84	9.43	9.54	8.89
8	1000		10.26	9.07	9.54	9.11
**TPC (sample 8) compared to original OMW, %**			96.34	85.16	89.58	85.54

Table S2. Antioxidant capacity of ethanol fraction (the concentration peak of desorption) measured by FRAP, ORAC, and DPPH assays.

Results are expressed as mean values of measurements in millimole Trolox equivalents (mM TE) (each sample was measured in triplicate).

	DPPH (mM TE)	FRAP (mM TE)	ORAC (mM TE)
Original OMW	1.74 ± 0.03	11.85 ± 0.08	61.00 ±7.20
Seplite LXA84	3.83 ± 0.00	85.51 ± 0.75	670.47 ±16.83
Seplite LXA88	3.77 ± 0.01	33.74 ± 0.67	257.68 ± 20.85
Seplite LXA816	3.50 ± 0.09	29.72 ± 0.85	203.99 ± 20.85
Amberlite XAD4	3.89 ± 0.00	50.56 ± 0.71	261.93 ± 18.72

Table S3. HPLC-determined concentrations of the main polyphenolic compounds in the original OMW and in selected 100 mL ethanolic desorption fractions collected at the peak of elution from adsorbent resins.

Values are expressed as mg L^{-1} and presented as mean values of duplicate measurements.

Polyphenolic Compound	Original OMW	Seplite LXA84	Seplite LXA88	Seplite LXA816	Amberlite XAD4
Hydroxytyrosol	675.96	6548.4	2792.9	1047.3	2077.9
3.4-hydroxybenzoic acid	179.96	1613.1	524.2	195.9	528.6
Tyrosol	235.30	1915.2	1032.5	n.d.	392.7
4-hydroxybenzoic acid	7.14	56.9	n.d.	n.d.	22.7
Vanillic acid	13.71	58.3	71.3	n.d.	n.d.
Caffeic acid	11.45	94.6	26.9	n.d.	17.9
t-Ferulic acid	1.25	1.62	10.3	n.d.	n.d.
Vanillin	0.82	27.2	n.d.	n.d.	n.d.
p-Coumaric acid	28.75	134.8	147.4	n.d.	n.d.
o-Coumaric acid	0.39	n.d.	n.d.	n.d.	n.d.

n.d. – not detected

Concentrations in the resin eluates refer to the HPLC-determined concentrations in the selected 100 mL concentrated ethanolic desorption fractions. These values were not converted to absolute masses. Absolute masses shown in Figure 6 were calculated separately as mass (mg) = concentration (mg L^{-1}) × volume (L).
